# HIV testing policies for migrants and ethnic minorities in EU/EFTA Member States

**DOI:** 10.1093/eurpub/ckt108

**Published:** 2013-08-05

**Authors:** Debora Alvarez-del Arco, Susana Monge, Ana M. Caro-Murillo, Oriana Ramírez-Rubio, Amaya Azcoaga-Lorenzo, Maria J. Belza, Yaiza Rivero-Montesdeoca, Teymur Noori, Julia Del Amo, the Study Working Group

**Affiliations:** 1 National Centre of Epidemiology, Instituto de Salud Carlos III, Madrid, Spain; 2 Division of Environmental and Reproductive Epidemiology, Biomedical Research Network on Epidemiology and Public Health (CIBERESP), Spain; 3 Department of Health Sciences. Universidad Rey Juan Carlos I, Madrid, Spain; 4 National School of Public Health, Instituto de Salud Carlos III, Madrid, Spain; 5 European Centre for Disease Prevention and Control (ECDC), Stockholm, Sweden

## Abstract

**Background:** In the context of an European Centre for Disease Prevention and Control (ECDC) research project, our objective was to describe current recommendations regarding HIV testing and counselling targeting migrants and ethnic minorities in the European Union/European Economic Area/European Free Trade Association (EU/EEA/EFTA) Member States. **Methods:** An on-line survey was conducted among 31 EU/EEA/EFTA Member States. The survey inquired on the existence of specific HIV testing and counselling recommendations or policies for migrants and/or ethnic minorities and the year of their publication. Additionally, we performed a review of national recommendations, guidelines or any other policy documents retrieved from an Internet search through the different countries’ competent bodies. **Results:** Twenty-nine (94%) country representatives responded the survey, and 28 documents from 27 countries were identified. National guidelines on HIV testing are heterogeneous and tailored, according to the epidemiological situation. Twenty-two countries identify migrants and four countries identify ethnic minorities as particularly vulnerable to HIV. Sixteen countries explicitly recommend offering an HIV test to migrants/ethnic minorities. Guidelines especially target people originating from HIV endemic countries, and benefits of HIV early detection are highlighted. HIV testing is not mandatory in any country, but some countries overtly facilitate this practice. **Conclusion:** Benefits of HIV testing in migrants and ethnic minorities, at both individual and community levels are recognized by many countries. In spite of this, not all countries identify the need to test these groups.

## Introduction

The HIV epidemic is a major public health problem in Europe^1^; 27 116 newly diagnosed cases of HIV infection were reported in 2010 by 28 countries of the European Union and European Economic Area (EU/EEA).^2^ Migrant populations, largely from Sub-Saharan Africa (SSA), represent a considerable proportion of AIDS cases and HIV infections, especially among women.^1^ Also, migrants are considered an important sub-population in the national response to HIV in most countries of the European Union and the European Free Trade Association (EU/EFTA).^3^ Definitions of migrants and ethnic minorities are heterogeneous between and within countries and throughout the different time periods,^4–7^ showing the lack of a standardized definition that is valid for any context. Migrants and ethnic minorities are not equivalent in term of populations although some migrants become part of established ethnic minorities.^5–7^

Most migrant groups, specially people of sub-Saharan African origin, have higher rates of late HIV diagnosis^8^ and, to some extent, the decline in AIDS incidence that followed the advent of combined Antiretroviral Therapy (cART) has not been observed.^1^^,^^9–11^ Barriers for migrants to access HIV testing and care can be placed at individual, health services, community and structural levels.^9^^,^^11–13^ Although migrants may share some of these barriers with disadvantaged native-born subjects, including ethnic minorities, those related to administrative and legal status affect migrants differently,^14^ establishing a population subgroup different to the native-born.^15^ Furthermore, some countries in the European context do not provide HIV care for people of uncertain legal status.^16^

The European Centre for Disease Prevention and Control (ECDC) issued its HIV testing Guidelines in December 2010 and recommended that all migrants from countries with high HIV prevalence should be offered an HIV test under the premises that testing should be voluntary, confidential and undertaken after previous informed consent.^17^ These guidelines acknowledge that the single biggest benefit of HIV testing is access to treatment and that the provision of cART should be the cornerstone of national HIV testing strategies. However, there may be vast differences in HIV testing policies between EU/EFTA country which are, ultimately, the ones that dictate local practice. We aim to describe recommendations and its rationale regarding HIV testing and counselling for migrants and ethnic minorities issued by the EU/ EFTA Member States up to the publication of the ECDC HIV testing Guidelines in December 2010.

## Methods

An online survey was conducted among 31 EU/EFTA Member States. Key informants were selected by ECDC within the competent health authorities (Ministries of Health and Public Health institutions) listed in the Appendix (Supplementary File S1). The survey inquired on the existence of specific HIV testing and counselling recommendations or policies for migrants and/or ethnic minorities and the year of their publication. Respondents were also asked to provide the most up-to-date document containing these recommendations. Various e-mail reminders were sent to encourage participation between February and September 2010. Overall, 29 (94%) countries responded. Data on participating countries are provided in the Appendix (Supplementary File S1).

We performed an additional search in the web pages of the different competent bodies of the EU/EFTA Member States (National AIDS Plans, Ministries of Health, Public Health Agencies, etc.) for national recommendations, guidelines or any other policy documents. Retrieved documents are listed in the Appendix (Supplementary File S2).

A data extraction form was designed and piloted. Teams of two independent researchers read the documents and extracted information on the publication date, issuing body and type of document (guidelines, health plan, piece of legislation, etc.). The data extraction form also contained information on the rationale for HIV testing at individual and community levels, on the definition of migrants and ethnic minorities, on whether their vulnerability to HIV infection was acknowledged, and/or if these populations were identified as groups to be offered HIV testing. In the cases where HIV test was recommended for migrants and ethnic minorities, information on the frequency and the site for HIV testing was recorded as well as recommendations for pre- and post-test counselling. Special attention was paid to whether the country recommended HIV testing on arrival of migrants and to any reference to the legal consequences of testing.

Additional collaboration of the national representatives was requested when the documents were in languages different to English, French, German, Italian, Portuguese or Spanish. However, in three central European countries, documents could not be reviewed and only responses from national key informants were used.

## Results

**Figure 1:**
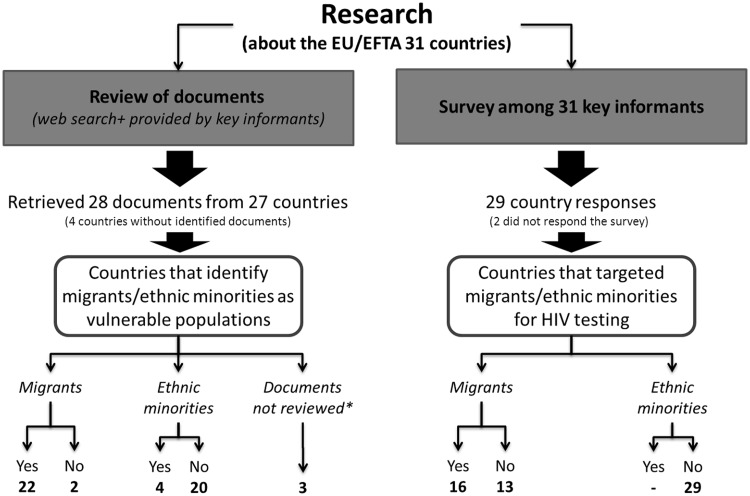
Research flow chart

Research flow chart is described in figure 1. Overall, 28 documents from 27 EU/EFTA Member States were identified: sixteen HIV/AIDS National Strategies, three HIV testing Guidelines, four miscellaneous documents about HIV testing recommendations, two internal working documents, one piece of legislation, one National Strategy on Communicable Diseases and one national communicable diseases surveillance bulletin.

### Rationale for early HIV testing

Documents from seven countries (Denmark, France, Norway, Portugal, Spain, Switzerland and the UK) discuss the benefits of early HIV diagnosis at individual and community levels when testing is linked to adequate referral to specialized services and treatment. Reduction in HIV viral replication and decreased HIV transmission from patients on treatment is presented as part of the rationale. Testing and counselling are also reported to be linked to positive behavioural change, although the French Guidelines remind this has only been documented for HIV-positive individuals. The UK Guidelines describe long pre-counselling as not necessary and recommends replacing it by brief pre-test information addressing the benefits of testing, its voluntary nature and the request of informed consent.

### Migrants and ethnic minorities as vulnerable populations for HIV

In these documents, migrants are mainly defined based on their region of origin. Overall, 22 of the 31 countries (71%) identify migrants as populations at risk for HIV infection (table 1) and specifically mention people from high HIV prevalence regions such as SSA and the Caribbean, and some specific groups from Eastern Europe, Asia and South America. Some documents only use the term ‘migrant’, and fewer documents refer to ethnic minorities. Only Bulgaria, Slovakia, Romania and the UK identify ethnic minorities as vulnerable to HIV. The Romanian Guidelines published in 2007 explicitly acknowledge the vulnerability of Roma people, given their high prevalence of risk contexts and behaviours; the Bulgarian HIV National Program also supports this, and the Slovakian program goes as far as to consider Roma community as part of marginalized groups coming from different ethnic or social environments. Black and minority ethnic groups (BME) are identified as vulnerable for HIV infection in the UK National Strategy although the National HIV Testing Guidelines, issued in 2008, refer to migrants rather than BME.

**Table 1:** Countries identifying migrants or ethnic minorities as vulnerable populations to HIV infection (from documents provided by National Representatives or found trough the web search)

Migrant/ethnic minorities identification as vulnerable populations to HIV	Countries
Identify migrants as vulnerable to HIV	Belgium, Bulgaria, Denmark, Finland, France, Germany, Iceland, Ireland, Italy, Lithuania, Luxembourg, Malta, Netherland, Norway, Poland, Portugal, Romania, Slovakia, Spain, Sweden, Switzerland and UK
Do not identify migrants as vulnerable to HIV	Cyprus and Slovenia
Identify ethnic minorities as vulnerable to HIV	Bulgaria, Slovakia^a^, Romania^b^ and UK^c^
Do not identify ethnic minorities as vulnerable to HIV	Belgium, Cyprus, Denmark, Finland, France, Germany, Iceland, Ireland, Italy, Lithuania, Luxembourg, Malta, Netherlands, Norway, Poland, Portugal, Slovenia, Spain, Sweden and Switzerland
No data (no documents provided by country representatives or found in web research)	Austria, Czech Republic, Hungary, Estonia, Greece, Latvia and Liechtenstein

^a^It considers some marginalized groups coming from different ethnic or social environments (Roma, homeless, refugees).

^b^Roma people are identified as part of disadvantaged communities.

^c^In the UK, BME are identified as HIV vulnerable in the National Strategy although the UK National Guidelines of HIV Testing 2008 does refer to migrants rather than BME.

Four documents (from France, Italy, Spain and the UK) mention the higher proportion of delayed HIV diagnosis in migrants, attributed to barriers to access health care such as stigma, lack of community support, isolation, racism and double discrimination for being an HIV-positive migrant. Cultural aspects, for instance religion and language, are described as additional barriers to access prevention and care. A disadvantaged socio-economic situation (poverty, low education, unemployment or poor working conditions) is also taken into account in a number of documents (from Bulgaria, Germany, Italy, Luxembourg, Romania and Spain) together with the higher vulnerability of some migrant women owing to low social status and double moral standards to judge female and male sexual behaviour. The French Guidelines and the Spanish Plan on HIV/AIDS describe how women may be exposed to gender violence, a source of vulnerability for HIV infection; women in abusive relationships have increased risk of HIV infection because of fear to oppose coercive sex.

### HIV testing recommendations addressing migrants and ethnic minorities

Even though 22 country key informants or national documents explicitly acknowledged that migrants were vulnerable to HIV infection (table 1), six of them—Germany, Ireland, Italy, Malta, Portugal and Spain—do not explicitly recommend HIV testing for migrants (table 2). Overall, 16 countries recommend HIV testing in migrant population whereas none recommends it in the case of ethnic minorities. Table 3 describes the groups specifically mentioned in each country’s document to be offered testing. Some countries refer generically to ‘migrant populations’ whereas others are more specific and limit the recommendation to migrants originating from high prevalence countries.

**Table 2:** Countries recommending HIV testing for migrants and ethnic minorities (information provided by National key Informants)

Recomendation of HIV testing for migrants/ethnic minorities	Countries
Recommend HIV testing for migrants	Belgium, Bulgaria, Denmark, Finland, France, Iceland, Lithuania, Luxemburg, Netherlands, Norway, Poland, Romania, Slovakia, Sweden, Switzerland and UK
Do not recommend HIV testing for migrants	Austria, Cyprus, Estonia, Germany, Greece^a^, Hungary, Ireland, Italy, Latvia, Malta, Portugal, Slovenia and Spain
Do not recommend HIV testing for ethnic minorities	Austria, Belgium, Bulgaria, Cyprus, Denmark, Estonia, Finland, France, Germany, Greece, Hungary, Iceland, Ireland, Italy, Latvia, Lithuania, Luxemburg, Malta, Netherlands, Norway, Poland, Portugal, Romania, Slovakia, Slovenia, Spain, Sweden, Switzerland and UK
No data	Czech Republic and Liechtenstein

^a^By the beginning of 2013, Greece will publish new HIV testing procedures that recommend HIV testing for migrants.

**Table 3:** Groups of migrants targeted for HIV testing

Country^*^	Groups mentioned
Belgium	Migrant population
Bulgaria	Refugees and asylum seekers; persons from high HIV prevalence countries and their sexual partners
Denmark	Persons from Africa, Asia, South America and Eastern Europe
France	High HIV endemic countries, especially SSA, Caribbean
Iceland	All migrants intending to stay for >1 year in Iceland as part of general health screening
Lithuania	Migrant populations
Luxembourg	People from SSA, Asia, Eastern Europe. Target groups of residents with foreign origin (especially Luxophon community)
Netherlands	High HIV prevalence countries (SSA, Surinam, Netherlands Antilles, South America, Eastern Europe and Asia); partners of people from HIV endemic area
Norway	People from high HIV endemic countries
Poland	All migrants are offered to have a voluntary HIV test on arrival as part of general health screening.
Slovakia	All migrants to have an HIV test on arrival as part of general health screening
Sweden	People from high endemic areas
Switzerland	People from countries with generalized epidemic
UK	People from high HIV prevalence countries

*By the beginning of 2013, Greece will publish new HIV testing procedures that recommends HIV testing in individuals originated from generalized epidemic countries (Sub-Saharan Africa) and men and women who report sexual contacts with individuals originated from high prevalence countries.

The recommended frequency of HIV testing for migrants is specified in documents from Denmark, France and the UK. Denmark recommends testing on the first contact with the health care system regardless of the reason, while France and the UK recommend systematic screening for people originating from regions of high HIV prevalence. French Guidelines also suggest repeating HIV testing every year for persons with multiple partners originating from sub-Saharan Africa and the Caribbean.

Documents from various countries (Belgium, Bulgaria, France, Luxembourg, Norway, Portugal, Romania, Spain, Switzerland and the UK) highlight the importance of identifying the most appropriate setting and the need to broaden the scope of health services performing the test to be able to scale up HIV testing in migrant populations. Accordingly, documents from Belgium, Bulgaria, France, Norway, Portugal, Spain, Switzerland and the UK state the benefits of HIV testing in community settings where people who would not be accessed through conventional services can be reached. In this regard, NGOs and Community-Based Organizations (CBOs) are settings explicitly identified as suitable. The use of rapid HIV tests in community settings is also mentioned in six documents (Bulgaria, France, Portugal, Spain, Switzerland and the UK) as being progressively accepted to increase HIV testing uptake in people who would not be reached otherwise.

### Legal consequences of HIV testing for migrants

Fear that disclosure of HIV status would affect migrant status and Visa application process is mentioned in Ireland as a deterrent to testing. The German Guidelines state migrants of uncertain status involved in a deportation process will not be deported if antiretroviral treatment is not available in their home country. In France, a law^18^ allows irregular migrants to obtain the residence permit if they are diagnosed with a serious disease as HIV and treatment is not accessible in their country of origin. Iceland, Poland and Slovakia offer HIV testing on arrival as part of general health screening claimed to provide early and appropriate HIV counselling, referral and care. In Slovakia, all foreigners staying for a period >3 months owing to studies or work are required to undergo an HIV test. An HIV-negative result is required for their sojourn permit, and they can be requested to undergo HIV testing by the police if they cannot provide a document confirming HIV negativity.

## Discussion

Although >68% of the policy documents from the 31 EU/EFTA Member States identify migrants as a vulnerable population for HIV infection, only 52% recommend HIV testing for migrant populations. The definitions and concepts of the terms migrants and ethnic minorities are extremely heterogeneous in the various policy documents retrieved.^6^^,^^7^ Some countries refer to migrants while others provide more detail—migrants from HIV endemic countries, migrants from Sub-Saharan Africa, etc. Ethnic minorities are not mentioned in general as groups most at risk for HIV, the UK, Bulgaria, Slovakia and Romania being the notable exceptions.

The overwhelming evidence of the benefits of HIV testing both at the individual and the community level described in the scientific literature is also mentioned in these policy documents.^19–23^ As all major international testing guidelines acknowledge, HIV testing on its own cannot be the final goal, and it is essential to link testing with care, support and treatment, ensuring a clear referral pathway for those who test positive.^17^^,^^24–26^ This is not the case across the region, especially for migrants of uncertain legal status.^16^ As the economic crisis has worsened across the European region, some countries have implemented a reduction in their expenditure through different policies like decreasing the total public spending in medical care or the volume and quality of care.^27^^,^^28^ Recent data from OECD show that health spending per person and as a percentage of GDP fell across the European Union in 2010.^28^ This is the first time that health spending has fallen in Europe since 1975. In Spain, health spending fell 0.9% in 2010, compared with an average annual growth rate of 4.6% between 2000 and 2009. Governments find themselves under pressure to protect funding for acute care and are cutting other expenditures such as public health and prevention programmes. In Spain, for example, measures in 2012 have been proposed to deny health care to migrants of uncertain legal status.^29^ Pérez-Molina and Pulido^30^ have carried out an analysis about the consequences of this measure and stated that the expected savings in the short term would be much lower than expected savings in the long term. They showed its impact on the country’s public health would be negative, increasing mortality and morbidity owing to communicable diseases and increasing spending in the medium and long term. Owing to this expertise initiative, finally the policy has not been implemented. This type of policy is thought to undermine health system goals^27^; therefore, comprehensive and coherent policies are needed to improve health.^31^^,^^32^

Unawareness of national HIV testing policies by health care professionals could be a barrier to put in practice recommendations and could explain^31^^,^^33^ the low and variable coverage of known groups at risk.^34^

The EU/EFTA is a heterogeneous area conformed by 31 countries with different cultural, socio-economic, political, legal and migratory contexts. Besides, there are important differences in the epidemiology of HIV between countries, as well as remarkable disparities in the health systems and social welfare structures. Because the absolute and relative contribution of migrants and ethnic minorities to the number of HIV and AIDS cases also varies greatly between different Member States, the legislation and the practices towards testing these groups for HIV must, necessarily, be different too. Also, it is likely that fear of further stigmatization may have deterred countries’ public health authorities to identify migrants as key populations for HIV testing. Nevertheless, migrants from high HIV prevalence settings should be included as a key group to be offered HIV testing and care in national policy documents as the first step to decrease the undiagnosed fraction among migrants in the EU region, provided that core principles of HIV testing programmes are guaranteed.

In analysing policies and recommendations from different countries, we observed inconsistencies within single countries. A number of countries have no guidelines for HIV testing; others are planning to develop them; and some are in the process of updating their HIV/AIDS Plans. This may explain the discrepancies between the information provided directly to us by the country representatives and the information we retrieved from the documents. Data provided by the key informants were invaluable, as it was a direct source of information, especially relevant in the case of countries with languages unknown by the research team.

Moving towards the normalization of HIV testing with universal strategies in selected contexts, as for example promoting universal HIV testing in some areas with high HIV prevalence rates, should not substitute HIV testing programmes for groups more at risk, such as migrants from HIV endemic countries. Nevertheless, a prioritization of resources based on cost-economic analyses and knowledge about implementation is needed.^35^

The various documents reviewed call to broaden the scope of health settings performing the HIV test, to strengthen proactive testing strategies, to put in place outreach programmes based on community approaches, like offering the test where people live, work or spend their leisure time, expanding testing hours and involving NGOs and Community Based Organizations through Point of care testing and rapid testing. The UK Health Protection Agency (now Public Health England) recommended giving support to primary care practitioners to assess the need for HIV testing in migrants.^36^ In fact, General Practitioners are also at the centre of HIV testing strategies in Germany, the Netherlands, Norway, Spain and Switzerland,^34^ and their perseverance has proved to be a key factor in increasing the uptake of HIV testing.^37^

The objective of this study was not to exhaustively assess to what extent mandatory HIV testing is applied to migrants. However, in reviewing policy documents, it can be concluded that policies in some countries may more or less inadvertently facilitate this practice. A number of international testing guidelines, particularly those from ECDC^17^ and WHO,^24^ strongly advice against mandatory HIV testing for migrants.

In summary, most documents consider migrants as disproportionately affected by HIV infection, and the benefits of early diagnosis are highlighted. National policy documents should recommend voluntary HIV testing for migrants as the first step to decrease the higher undiagnosed fraction in this population in the EU/EFTA region. Although national HIV testing policies are heterogeneous across the countries in the European region, HIV testing approaches must rely on the pillar that testing must be linked to care as clearly stated in the ECDC HIV testing Guidelines.^17^ The fact that there are countries which do not provide universal HIV prevention, treatment and care for migrants of uncertain legal status challenges the ethics and the effectiveness in the application of the pro-active HIV testing and are a deterrent for the control of an important public health problem in the region.

## Supplementary data

Supplementary data are available at *EURPUB* online.

Supplementary Data

## Appendix 1

### Study working group

Álvarez-del Arco D, Monge S, Caro-Murillo AM, Ramírez-Rubio O, Azcoaga A, González C, Hernando V, Alejos B, Pérez-Cachafeiro S, Río I, Rivero-Montesdeoca Y, Belza MJ, Bolúmar F, Noori T and Del Amo J.
