# Supplementary material for: HIV testing policies for migrants and ethnic minorities in EU/EFTA Member States
Source: Eur J Public Health. 2013 Aug 5;24(1):139–44. doi: 10.1093/eurpub/ckt108 (PMC3901314; doi:10.1093/eurpub/ckt108)
Supplement: Supplementary Data [file supp_24_1_139__index.html]

HIV testing policies for migrants and ethnic minorities in EU/EFTA Member States — HIV testing policies for migrants and ethnic minorities in EU/EFTA Member States — Supplementary Data 

# HIV testing policies for migrants and ethnic minorities in EU/EFTA Member States

## 

files

**Files in this Data Supplement:**

- Supplementary Data - docx file
- Supplementary Data - docx file
