# Supplementary material for: HIV testing policies for migrants and ethnic minorities in EU/EFTA Member States
Source: Eur J Public Health. 2013 Aug 5;24(1):139–44. doi: 10.1093/eurpub/ckt108 (PMC3901314; doi:10.1093/eurpub/ckt108)
Supplement: Supplementary Data [file supp_ckt108_ejph-2012-12-om-0924-File006.docx]

**Supplementary file 1. List of National representatives**

| **Country** | **Country representative** | **Position** | **Institution** |
| --- | --- | --- | --- |
| **Austria** | Jean-Paul Klein | Technical expert HIV/AIDS, tuberculosis, immunisation | *Ministry of Health* |
| **Belgium** | André Sasse | Epidemiologist | *Scientific Institute of Public Health* |
| **Bulgaria** | Tsvetana Yakimova | Chief Expert | *Ministry of Health* |
| **Cyprus** | Anna Nouska | AIDS Programme Manager | *Ministry of Health* |
| **Denmark** | Susan Cowan | Medical Consultant, Public Health | *Department of Epidemiology, Epidemiology Division* |
| **Estonia** | Aljona Kurbatova | Researcher | *Infectious Diseases and Drug Abuse Prevention Department, National Institute for Health Development* |
| **Finland** | Henrikki Brummer | Research Manager | *HIV Laboratory, National Institute for Health and Welfare* |
| **France** | Caroline Semaille | Medical Epidemiologist | *Institut de Veille Sanitaire* |
| **Germany** | Osamah Hamouda | Researcher | *Federal Ministry of Health* |
| **Greece** | Georgios Nikolopoulos | Epidemiologist | *HIV Infection Office, Hellenic Centre for Diseases Control and Prevention* |
| **Hungary** | Maria Dudas | Epidemiologist | *Hungarian National Centre for Epidemiology* |
| **Iceland** | Sigurlaug Hauksdóttir | Social Consultant | *Centre for Infectious Disease Control, Directorate of Health* |
| **Ireland** | Aidan O'Hora | Consultant in Public Health Medicine | *Health Protection Surveillance Centre* |
| **Italy** | Anna Maria Luzi // Anna Colucci | Researcher, Scientific Chief, Psycho-Socio-Behavioural Research, Communication and Training Operating Unit, and Italian National Focal Point for Infectious Diseases and Migrant // Researcher, Research Coordinator, Psycho-Socio-Behavioural Research, Communication and Training Operating Unit. | *Department of Infectious, Parasitic and Immunomediated Diseases, National Institute of Health* |
| **Latvia** | Inga Upmace | Head of AIDS Programme Department | *Infectology Centre* |
| **Lithuania** | Oksana Strujeva | Epidemiologist | *HIV/AIDS/STI and Hepatitis Epidemiological Surveillance Department, Centre for Communicable Diseases and AIDS* |
| **Luxemburg** | Robert Hemmer | Chairman, National AIDS Committee | *National Service of Infectious Diseases, Centre Hospitalier de Luxembourg* |
| **Malta** | Jackie Melillo | Public Health Physician | *Infectious Disease Prevention and Control Unit, Health Promotion and Disease Prevention Directorate, Ministry for Social Policy* |
| **Netherlands** | Eline Op de Coul | Epidemiologist | *Centre for Infectious Disease Control, National Institute for Public Health and the Environment* |
| **Norway** | Hans Blystad | Deputy Director | *Department of Infectious Disease Epidemiology, Norwegian Institute of Public Health* |
| **Poland** | Anna Marzec-Bogusławska | Director | *National AIDS Centre* |
| **Portugal** | Sónia Dias | Assistant Professor, International Health Department | *Institute of Hygiene and Tropical Medicine, University of Lisbon* |
| **Romania** | Mariana Mardarescu | Consultant in Infectious Diseases, Head of Paediatric and Adolescents Immunosuppression Department, Coordinator Compartment for Monitoring and Evaluation of HIV/AIDS | *National Institute for Infectious Diseases "Prof. Dr. Matei Bals" of Bucharest* |
| **Slovenia** | Mario Poljak | Head of Laboratory for Molecular Microbiology and Slovenian HIV/AIDS Reference Centre | *Institute of Microbiology and immunology, Faculty of Medicine, University of Ljubljana* |
| **Slovakia** | Danica Stanekova//Alexandra Zampachova | Head, National Reference Center for HIV/AIDS Prevention // Epidemiologist | *National Reference Center for HIV/AIDS Prevention//Public Health Authority* |
| **Spain** | Olivia Castillo | Head, Prevention and Coordination, Department for National AIDS Strategy | *Ministry of Health and Social Policy* |
| **Sweden** | Robert Jonzon | Senior Programme Officer, National Coordination of HIV/STI Prevention Unit | *National Board of Health and Welfare* |
| **Switzerland** | Luciano Ruggia | Project Manager, International Affairs | *Federal Department of Home Affairs, Federal Office of Public Health, Division of Communicable Diseases. Prevention and Promotion Section* |
| **UK** | Valerie Delpech | Consultant Epidemiologist and Head of HIV and AIDS Reporting Section | *Centre for Infections, Health Protection Agency* |
