# Supplementary material for: HIV testing policies for migrants and ethnic minorities in EU/EFTA Member States
Source: Eur J Public Health. 2013 Aug 5;24(1):139–44. doi: 10.1093/eurpub/ckt108 (PMC3901314; doi:10.1093/eurpub/ckt108)
Supplement: Supplementary Data [file supp_ckt108_ejph-2012-12-om-0924-File007.docx]

**Supplementary file 2. List of documents used (provided by National informants or found in web search)**

| **Country** | **Document** | **Type** | **Language** |
| --- | --- | --- | --- |
| **Belgium** | *Les strategies concertées du secteur de la prévention des IST/SIDA en Communauté française de Belgique; Les migrants (2009). Proactief HIV-testen en –counselen voor subsah (2010)* | Recommendations issued by French and Flemish Communities | French |
| **Bulgaria** | *National Programmes for Prevention and Control of HIV and STIs (2008-2015)* | HIV National strategy | Bulgarian |
| **Cyprus** | *Strategic plan 2004 – 2008 HIV/AIDS Cyprus (in drafting a new version)* | HIV National strategy/National plan | English |
| **Czech Republic** | *Národní program řešení problematiky HIV/AIDS v České republice v období let 2008 – 2012 (dále jen program)* | HIV National strategy/National plan | Czech |
| **Denmark** | *EPI-news. National surveillance of communicable diseases. No. 46, 2009* | Communicable diseases unit communiqué | English |
| **Estonia** | *Eesti riiklik HIV ja AIDSi strateegia aastateks 2006 – 2015* | HIV National strategy/National plan | Estonian |
| **Finland** | *Suomen hiv-strategia 2013-2016 Pakolaisten ja turvapaikanhakijoiden infektio-ongelmien ehkäisy.* | Recommendations | Finnish |
| **France** | *HIV screening in France – Public health guidelines, 2009* | Guide | French |
| **Germany** | *Action Plan to implement the Strategy of the Federal Government to fight HIV/AIDS, 2007* | HIV National strategy/National plan | English |
| **Hungary** | *Nemzeti AIDS stratégia 2004 –2010* | HIV National strategy/National plan | Hungarian |
| **Iceland** | *Procedures for the Medical Examination of Immigrants to Iceland. 2007* | Law | English |
| **Ireland** | *HIV and AIDS Education and Prevention Plan 2008 – 2012* | HIV National strategy/National plan | English |
| **Italy** | *Aggiornamento delle conoscenze sulla terapia dell’infezione da HIV -Documento Complementare su Specifiche Materie, Febbraio 2008* | Work document | Italian |
| **Lithuania** | *National HIV / AIDS and STI Prevention and Control Programme for 2010- 2012* | HIV National strategy/National plan | English |
| **Luxembourg** | *Strategie et plan action sida 2006-2010* | HIV National strategy/National plan | French |
| **Malta** | *Communicable disease control strategy for Malta. 2003* | National strategy | English |
| **Netherland** | *Regeling. Aanvullende Curatieve Soa-bestrijding. 2008* | Work document | Dutch |
| **Norway** | *Strategy: Acceptance and coping. National HIV strategy 2009-2014 (2009)* | HIV National strategy/National plan | English |
| **Poland** | *Polskie Towarzystwo Naukowe AIDS. REKOMENDACJE PTN AIDS 2006 Zasady Opieki Medycznej nadOsobami Zakażonymi HIV* | Recommendations | Poland |
| **Portugal** | *Programa nacional de prevenção e controlo da infecção vih/sida. 2007-2010* | HIV National strategy/National plan | Portuguese |
| **Romania*** | *National strategy for surveillance, control and prevention of HIV/AIDS 2004-2007* | HIV National strategy/National plan | English |
| **Slovakia** | *ODBORNÉ USMERNENIE na zabezpečenie prevencie infekcie spôsobenei vírusom imunitnej nedostatočnosti človeka v Slovenskej republike.* | HIV National strategy/National plan | Slovak |
| **Slovenia** | *Strategija prepre evanja in obvladovanja okužbe s hiv za obdobje 2010–2015* | HIV National strategy/National plan | Slovene |
| **Spain** | *Plan Multisectorial frente a la infección por VIH y el sida España 2008-2012* | HIV National strategy/National plan | Spanish |
| **Sweden** | *Nationell strategi mot hiv/aids och vissa andra smittsamma sjukdomar. National Strategy to Combat HIV/AIDS and Certain Other Communicable Diseases (2007)* | HIV National strategy/National plan | Swedish/ English |
| **Switzerland** | *Recommandations de l’OFSP sur le conseil et le dépistage volontaire du VIH (VCT) au moyen d’un test VIH rapide dans les centres de dépistage* | Guide | French |
| **UK** | *UK National Guidelines for HIV Testing 2008* | Guide | English |

(*) The National HIV/AIDS Strategy 2012-2016 is in process of approval)
